# Machine learning techniques on homological persistence features for prostate cancer diagnosis

**DOI:** 10.1186/s12859-022-04992-5

**Published:** 2022-11-12

**Authors:** Abbas Rammal, Rabih Assaf, Alban Goupil, Mohammad Kacim, Valeriu Vrabie

**Affiliations:** 1 Statistics and Computer Sciences Department, Faculty of Science, Lebanese University, Beirut, Lebanon; 2 UMR CNRS 6158 LIMOS: Laboratoire d’Informatique, de Modélisation et d’Optimisation des Systèmes, Institut Henri Fayol, École des Mines de Saint-Etienne, Saint-Étienne, France; 3 Department of Mathematics, Faculty of Arts and Sciences, Holy Spirit University of Kaslik, Jounieh, Lebanon; 4 CReSTIC EA 3804, Université de Reims Champagne Ardenne, 51097 Reims, France

**Keywords:** Prostate cancer diagnosis, Gleason scores, Topological data analysis, Homology persistence, Supervised learning

## Abstract

The rapid evolution of image processing equipment and techniques ensures the development of novel picture analysis methodologies. One of the most powerful yet computationally possible algebraic techniques for measuring the topological characteristics of functions is persistent homology. It's an algebraic invariant that can capture topological details at different spatial resolutions. Persistent homology investigates the topological features of a space using a set of sampled points, such as pixels. It can track the appearance and disappearance of topological features caused by changes in the nested space created by an operation known as filtration, in which a parameter scale, in our case the intensity of pixels, is increased to detect changes in the studied space over a range of varying scales. In addition, at the level of machine learning there were many studies and articles witnessing recently the combination between homological persistence and machine learning algorithms. On another level, prostate cancer is diagnosed referring to a scoring criterion describing the severity of the cancer called Gleason score. The classical Gleason system defines five histological growth patterns (grades). In our study we propose to study the Gleason score on some glands issued from a new optical microscopy technique called SLIM. This new optical microscopy technique that combines two classic ideas in light imaging: Zernike’s phase contrast microscopy and Gabor’s holography. Persistent homology features are computed on these images. We suggested machine learning methods to classify these images into the corresponding Gleason score. Machine learning techniques applied on homological persistence features was very effective in the detection of the right Gleason score of the prostate cancer in these kinds of images and showed an accuracy of above 95%.

**Supplementary Information:**

The online version contains supplementary material available at 10.1186/s12859-022-04992-5.

## Introduction

Prostate cancer (PCa) is the second most frequent malignancy in males worldwide and the fifth leading cause of death from cancer. About 75 percent of patients detected are older than 65 years, and it is extremely unusual in children and teenagers. In most cases, the incidence and death rates rise with age [1–3].The biggest risk factors are age and family history [4].

Considering 1.2 million additional instances of prostate cancer diagnosed each year [5], a high incidence-to-mortality ratio, and the danger of overdiagnosis and overtreatment [6, 7], proper prognostic evaluation is critical. About 50 years ago, Dr. Donald Gleason developed a prostate cancer scoring system that is based on histopathological findings. With many revisions, this present system keeps its validity. The Gleason score is the most effective prognostic predictor for prostate cancer (PCa) patients, as determined by a pathologist after microscopic study of disease morphology. This score is made up of two component scores that correspond to the tissue's most and second most prevalent grades. Each partial score is a letter grade between 1 and 5. Gleason 1 represents the best differentiated, that keeps the shape of the tissue and its components. It is associated with the best prognosis. Whereas Gleason 5 is the lowest differentiated, where we remark a big change in the shape of the tissue and its components and is associated with a bad prognosis.

The International Society of Urological Pathology published new revisions in 2005 and 2014. The 2005 ISUP changes to the Gleason scoring system for prostate cancer classify patients properly based on pathological processes and summary biochemical results, However, a revision in reporting is required to better depict tumor behavior while maintaining the Gleason system's essence [8]. The new Gleason proposal should include prognostic grade groups, which were as shown in: Gleason score 6 (prognostic grade group I); Gleason score 3 + 4 = 7 (prognostic grade group II); Gleason score 4 + 3 = 7 (prognostic grade group III); Gleason score 4 + 4 = 8 (prognostic grade group IV); and Gleason score 9–10 (prognostic grade group V). Noting that the first score represents the most dominant pattern in the tissue while the second score, the second most dominant score. For example, 4 + 3 says that the most dominant score is 4 and the second most dominant is 3. This why the prognostic grade group II differs from III [9, 10].

**Fig. 1:**
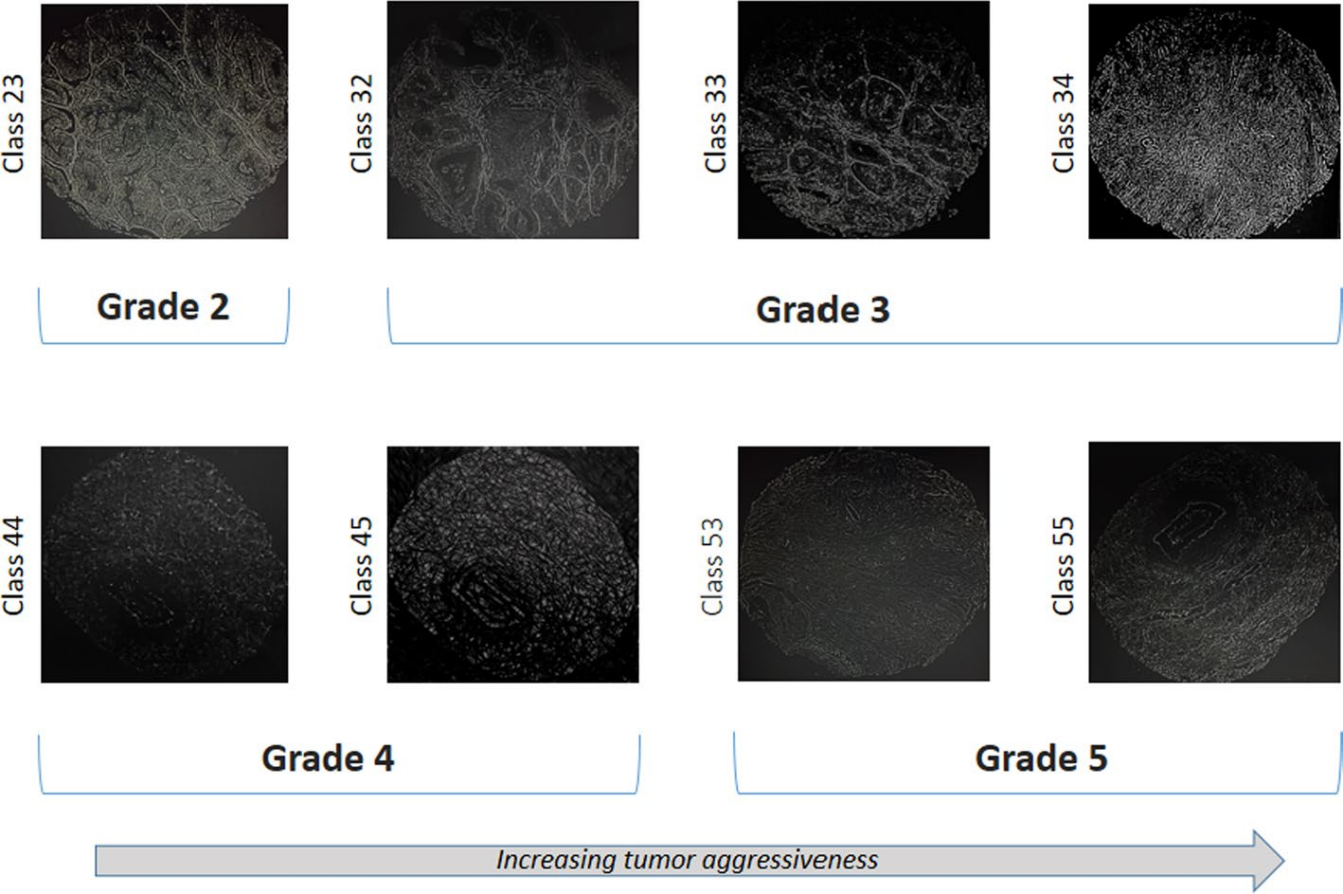
Four Gleason scores of the modern Gleason grading system and their corresponding classes. A higher grade number indicates higher aggressiveness of the cancer

Prostate cancer treatment is mostly determined by the biopsies Gleason score. Figure 1 shows eight distinct patterns displayed in our study. To date, prostate biopsy suggests that a single way to determine the cancer's grade is by using imaging techniques. Major advancements in imaging technology have improved disease detection and localization [11]. Consequently, The Gleason grading is an important component in prostate diagnosis since it has a high association with the severity of the disease and the patient's chance of survival. It also aids the pathologist in determining the best treatment options for the patients [12].

At the level of machine learning, there were many studies and articles recently witnessing the combination between prominence of Gleason score on PCa diagnosis using machine learning techniques. The major strength of machine learning is its capacity to evaluate and use massive amounts of data considerably more quickly than humans can using traditional statistical studies. As a result, it's not surprising that its expanding importance in radiology has coincided with radiomics' growing importance and potential in research. This is another rapidly growing field that permits large amounts of quantitative data to be extracted from medical images [13]. These big datasets were examined for clinical information such as correlations with other biomarkers, patient prognosis, and treatment outcomes [14, 15]. In the field of PCa, there is a lot of interest in examining the use of ML-based computer-assisted diagnostic (CAD) software. For example, Kwak et al. [16] have shown that radiomics and machine learning may be used to analyze different tissues and cellular densities in the prostate gland to aid in PCa detection. According to Ginsburg and al [17]_,_ different ML prediction models for the transition and peripheral zones in a tissue should be constructed, because lesions and normal prostatic tissue have different imaging properties in these zones. In the transition zone, ML can also be used to distinguish stromal benign prostatic hyperplasia from PCa. This diagnosis might be difficult, particularly with tiny lesions. Using either linear regression or the SVM classifier, statistical analysis of previously established quantitative variables (ADC maps, shape, and picture texture) exhibited high accuracy categorization of microscopic malignant lesions from benign ones using either linear regression or the SVM classifier. Using either linear regression or SVM classifiers, accuracy in the classification of tiny malignant tumors from benign lesions was achieved [18].

Topological data analysis (TDA) is a method that captures the geometric shape (i.e., coarse scale, global, nonlinear geometric properties) of high-dimensional data sets using algebraic topology ideas. TDA has been applied to a wide range of data, from molecular to population-level data, with great success [19, 20]. TDA is ideally adapted to the problem of quantifying the architecture of prostate cancer from a protastic standpoint. For histological classification, the Gleason grading system depends solely on the detection of architectural patterns generated by groupings of cancer cells and nearby stroma. Even in higher-dimensional space, TDA allows you to grasp the shape of data and hence architecture. Recently, persistent homology (PH) has been developed as a new multiscale representation of topological features. Among the algebraic topology tools, persistent homology is one of the most powerful yet computationally feasible algebraic techniques for measuring the topological features of functions.

In our work we propose to study the Gleason score on some glands issued from the SLIM technique. This technique reveals the intrinsic contrast of cell structures and, in addition, renders quantitative optical path-length maps across tissues. Persistent homology features are computed on these images. ML techniques applied on homological persistence features can be very effective in the detection of the right Gleason score of the prostate cancer in these kinds of images. Thus, our goal is to procreate a better grasp for pathologists in the Gleason grading system through the combination of topological data analysis with machine learning algorithms. In [21], the authors described a method that discriminate between the Gleason scores 2.3 and 4 using persistent homology features and unsupervised learning techniques. Our method works in the same area, but it’s more efficient on the level of the classification of degrees of Gleason score as classes and sub-classes. It can detect more scores. In addition, more methods of machine learning are applied in our article thus it opens a wider perspective and allows more advanced results. The prostate glands images are classified into subclasses of Gleason Score. We extract glands from the images using ImageJ SOftware then we compute topological features over windows in these glands. After, we apply machine learning methods to predict the classes of each window, which allow us to know the class of each window and eventually each Gland. Then we calculate the precision metric over all the Gland in each image.

## Mathematical methods

### Topological data analysis

TDA extracts an important set of topological features from high-dimensional data sets that complement geometric and statistical features, which offers a different perspective for machine learning. Features generated from traditional topological models like cell complexes on the other hand, preserve the global intrinsic structure information, but they tend to reduce too much structure information and are rarely used in quantitative characterization. In [22] for example, the authors elaborate the idea of Computational Topology that allows the implementation of topological tools into well-established algorithms to manipulate data. In this work, we focus on the application of persistent homology, one of the most widely studied and applied TDA tools.

### Persistent homology

**Fig. 2:**
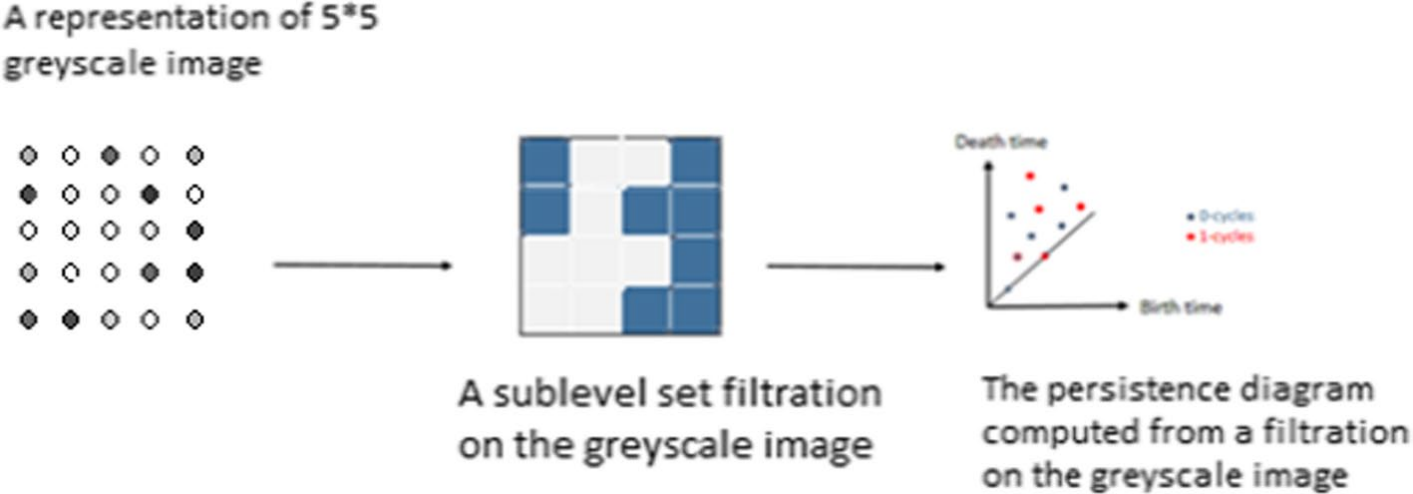
The usual use of persistence in TDA. A filtered complex on the top of the dataset is built. The persistent complex is then computed and shown on a persistence diagram

PH is one of the most widely studied and applied TDA tools. Unlike commonly used computational homology which results in truly metric-free or coordinate-free representations, persistent homology is able to embed geometric information into topological invariants so that the “birth” and “death” of isolated components, circles, rings, loops, pockets, voids or cavities at all geometric scales can be monitored by topological measurements. The authors in [23] and [24] explain in details how the persistent homology is computed through the nested topological spaces. In [25], the authors introduce an efficient persistent homology computation method using the Morse theory to build the nested topological spaces to get a scheme called filtration. Compared with traditional computational topology [26] and/or computational homology, persistent homology inherently has an additional dimension, namely, the filtration parameter, which can be utilized to embed some crucial geometry or quantitative information into the topological invariants (see Fig. 2). This figure shows a 5 * 5 example of a greyscale image then it shows one sublevel set built from this image through the process of filtration. The pixels of the image represent points in our space. We connect these points by edges and then 2D squares through the scheme of filtration of sublevels. You can see in the second step an example of a one sublevel set of these sets. At the end, a persistence diagram is computed and showing the lifetime of persistent homology classes. Barcode representation has been proposed for the visualization of topological persistence [27], in which various horizontal line segments or bars are utilized to represent the persistence of the topological features.

The workflow of the proposed topological features computation of a gray scale image begins with the concept of “spatialization”, transforming the image into spaces. A combinatorial representation for the spaces and the linearization of this representation permit to compute homological groups. A scheme of nested sequences of spaces called filtration permit to compute persistent homology groups, which are the homology groups that persist to variation of spaces during this scheme. Each of these steps are described in extent below [28].

*Spatialization* The input image is viewed as a continuous function $f$ from the domain $D \subset {\mathbb{R}}^{2}$ into the real line ${\mathbb{R}}$, i.e. $f:D \to {\mathbb{R}}$. This point of view is correct for grayscale images and thus several spaces can be defined regarding $f$. The sublevel sets are given by all points of the domain whose value does not exceed a level *a:*$U_{a} = f^{ - 1} \left[ { - \infty ;a} \right]$. The sublevel sets are ordered by their level *a* under inclusion, hence $U_{a} \subset U_{b}$ when $a < b < \ldots < z$*..* This permits to define the filtration as the nested sequence of spaces.

1 $$\emptyset \subset U_{a} \subset U_{b} \subset \cdots \subset U_{z} \subset D$$

*Combinatorial representation* The spaces under study are mathematically well defined but are not suited for algorithmic calculation. Therefore, spaces are decomposed into cells. The set of all cells and the gluing information provided by its boundaries are called the cell complex [29]. This is particularly suited in the case of grayscale image: a pixel is considered as a point, an edge represents the connection between neighborhood pixels horizontally and vertically. A square is formed from four neighbor edges. The image viewed as a function gives a value for each cell of its domain representation. For example, $f\left( x \right)$ is the grayscale value assigned to a pixel $x$. The value of an edge is the maximum of values of surrounding pixels, while a square’s value is the maximum of values of incoming edges. According to this procedure, the sublevel sets obtained by selecting the cells whose value is below a constant level $a$ is necessarily a sub-complex. It means that if a $k$-cell is in the complex, its boundary is also in the complex. This evaluation of cells implies that the filtration of spaces (1) has an equivalent filtration of complex along the level $a$ that preserves the inclusion property.

*Linearization* Even if it is possible to develop the following theory for general coefficients, we limit our work over ${\mathbb{Z}}_{2}$ for the sake of simplicity. The complex previously built gives birth to several vector spaces $C_{k}$ that are finite formal sums of $k$-cells. We call the elements of $C_{k}$ a $k$-chain_76_. That is,$c = \sum a_{i} \sigma_{i}$, where the $\sigma_{i}$ are the $k$-cells and the $a_{i}$ are the coefficients in ${\mathbb{Z}}_{2}$. Its boundary operator is $\partial_{k} c = \sum a_{i} \partial k\sigma_{i}$, where $\partial_{k} \sigma_{i}$ represents the boundary of the $k$-cell $\sigma_{i}$ and it’s the sum of the boundaries of its cells. Hence, by taking the boundary function we map a $k$-chain to a ($k - 1$)-chain. We write this homomorphism as $\partial_{k} : C_{k} \to C_{k - 1}$. A chain complex is a sequence of chain groups connected by boundary homomorphisms such that $\partial_{k - 1} \partial_{k} = 0$ for all dimensions k:

2 $$\ldots \to ^{{\partial_{k + 2} }} C_{k + 1} \to ^{{\partial_{k + 1} }} C_{k} \to ^{{\partial_{k} }} C_{k - 1} \to ^{{\partial_{k - 1} }} \ldots$$

*Homology* Homology is an algebraic and topological tool to detect connectivity of topological spaces. Boundary less $k$-chains are meaningful and form a subgroup of $C_{k}$ that we call the $k$-th cycle group $Z_{k}$:

3 $$Z_{k} = \{ x \in C_{k} |\partial_{k} x = 0\} = ker \partial_{k}$$

Among these cycles, we consider the ones that surround chains. They form a subgroup called the $k$-boundary group $B_{p}$.

4 $$B_{k} = \{ x \in C_{k} | \exists y \in C_{k + 1} , x = \partial_{k + 1} y\} = im \partial_{k + 1}$$

The k-th homology group $H_{k}$ is defined as the quotient group *Z*_*k*_/*B*_*k*_*.* It’s the group of non-bounding cycles*.* The homology group $H_{k}$ keeps the count of essentially different cycles that are interesting by distributing all cycles into equivalent classes. An element of $H_{k}$ gathers together equivalent cycles, which can be deformed continuously one onto the other. In other words, two cycles are equivalent if their difference is a boundary. In addition, the dimension of $H_{k}$ is called the $k$-th Betti number $\beta_{k}$. The Betti numbers in dimensions 0, 1, and 2 are the number of connected components, tunnels, and voids of the complex, respectively. Because of the linearity, homology groups $H_{k}$ can be easily computed by standard matrix manipulations given a combinatorial representation of the chain complex.

*Persistent homology* Persistent homology comes from the ideas of filtration and the functoriality of homology described above. Let $K = \left\{ {\sigma_{1} , \ldots ,\sigma_{i} } \right\}$ a cell complex of dimension d. We assume an ordering on the cells such that for each $i \le n$, $K_{i} = \left\{ {\sigma_{1} , \ldots ,\sigma_{i} } \right\}$ is a cell complex. The chain $\emptyset = K_{0} \subset K_{i} \ldots \subset K_{n} = K$ is a filtration of $K$. In our case, such a filtration is defined according to a function $f : K \to {\mathbb{R}}$ that orders the cells of $K$ by function value. In addition, by tracking the topological evolution of this filtration using homology, we get a sequence of homology groups that are connected by linear maps induced by inclusions:

5 $$H\left( {K_{0} } \right) \to H\left( {K_{1} } \right) \to \ldots \to H\left( {K_{i} } \right) \to \ldots \to H\left( {K_{n} } \right)$$

**Fig. 3:**
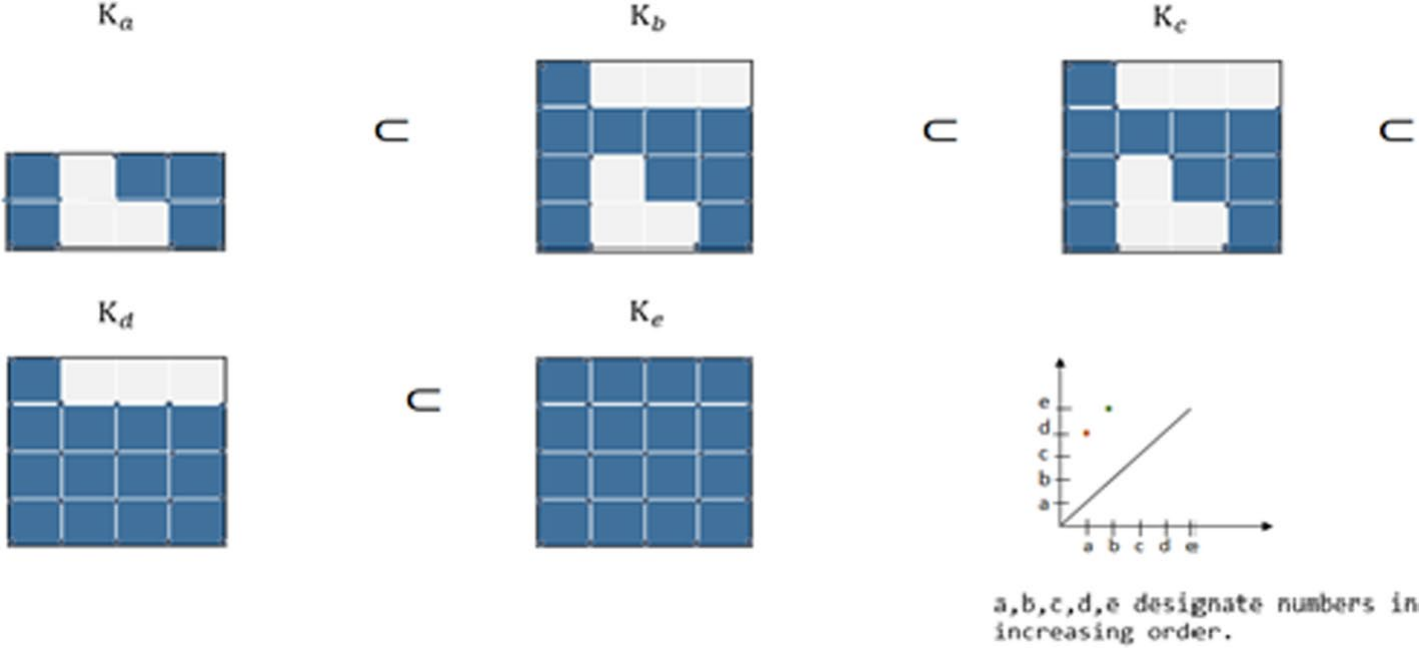
Filtration scheme from K_a_ to K_e_ showing the appearance and disappearance of homology groups, the first from *K*_*a*_ to* K*_*d*_, the second from *K*_*b*_ to* K*_*e*_ and the associated persistence diagram

Noting that these linear transformations are the maps associated with the inclusions of cell complexes [29]. Persistent homology tracks the appearance of classes in this sequence. As we go from $K_{i - 1}$ to $K_{i}$, we gain new homology classes and we lose some (see Fig. 3). In this figure, a two homology classes of dimension one appeared at times a and b, then they disappeared at times d and e respectively. The associated persistence diagram of dimension one shows two pints that indicate the time of appearance and disappearance of each homology class from $K_{a}$ to $K_{b}$.

*Computation of persistent homology* We can compute the homology $H_{p} (K_{i} )$ for all sublevel sets $K_{i}$ of (1) in order to depict the evolution of the number of topological features of an image. However, we lose the information concerning the evolution of each particular cycle. Indeed, a cycle may emerge at a given level $i$ and die further at the level $j$. Recording the “life duration” of each characteristic cycle is more informative than recording the evolution of Betti numbers. Along the procedure of filtration by intensity, adding a new cell $\sigma_{j}$ of intensity j and dimension k can contribute to two results. Either it “creates” a new homology class or a void or we call $\sigma_{j}$ a "creator", or an already existing homology class $\sigma_{i}$, created at intensity i, where i < j and of dimension k-1 can also be filled and thus "destroyed" and we call $\sigma_{j}$ a "destroyer". Therefore, the life duration of the homology class will be equal to the intensity difference between destroyer and creator, j-i. That means that it is given by the difference between the death time and its birth time along the filtration. The persistence, and its algorithm [30], gives this recording of the evolution of cycles along the level. The evolution of lifetimes of 0-cycles and 1-cycles can be represented using a persistence diagram or a barcode with respect to filtration time.

### Persistent homology based machine learning

**Fig. 4:**
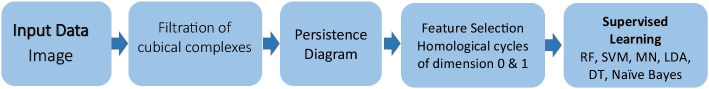
The flowchart for PHML modeling. For different data types, certain simplicial complexes are considered. With a suitable filtration parameter, PH analysis can be implemented by a designed software (PHAT). The result from the PH is transformed into feature vectors, which is further combined with supervised methods from machine learning

The persistent-homology-based machine learning (PHML) models have been used in various areas, including image analysis. The essential idea for PHML is to extract topological features from the data using PH, and then combine these features with machine learning methods, including both supervised learning and unsupervised learning approaches. As illustrated in (Fig. 4), PHML in computation can be divided into four steps, i.e., simplicial complex construction, PH analysis, topological feature extraction and topology-based machine learning. In [31] the authors describe and develop, WSI-GTFE a method that combines TDA and Graph Neural Network in order to identify and quantify key pathogenic information pathways to capture macro and micro architecture of histology images. In [32] the authors explain how a method that improves modern image classification techniques by considering topological features gave quite accurate results in classification of images using deep learning. Recently, the authors in [33] introduces a new method that talks about the fusion of TDA and Deep learning features for COVID-19 detection from chest X-Ray images.

The first step is to construct simplicial complex (or complexes) from the studied data. In topological modeling, we may have various types of data, including functional data, point cloud data, matrixes, networks, images, etc. Depending on the type of studied data, cubical simplex models can be considered. The cubical homology theory has been developed for a long time, especially for its application to image analysis. The decomposition of images and position of pixels make the use of the cubical complex the most appropriate one for the topological spaces. In [34] and [35] the authors give a detailed explanation on the construction of cubical complexes from grayscale images and how the filtration scheme is built from this topological complex. The second step is the PH analysis. In PH, algebraic tools, such as quotient group, homology, exact sequence, etc. are used to characterize topological invariants, including connected components, circles, rings, channels, cavities, voids, etc. Unlike traditional topological models, which capture only the intrinsic structure information and tend to ignore all geometric details, PH works as a multiscale topological representation and enables to embed geometric information back into topological invariants. This is achieved through a new idea called filtration. With a suitable filtration process, the persistence of topological invariants can be calculated. Roughly speaking, the persistence tells you the geometric size of the invariant. Different softwares are currently available for PH analysis of different data structures. We use PHAT, persistent homology algorithm toolbox introduced and described in [36]. It ensures a fast way to calculate persistent homology classes on the images. The third step is to extract meaningful topological features from PH results. The PH results are usually represented as (persistent barcode) PBs or (persistence diagram) PD. The third step is to extract meaningful topological features from PH results. The PH results are usually represented as (persistent barcode) PBs or (persistent diagram) PD. Neither of these representations can be used directly as input for machine learning models. Therefore, we need to transform the PH results into representations, which can be easily incorporated into machine learning models. The fourth step is to generate topological feature vectors from PH results. Binning approach is a used approach to discretize the PB or PD into a feature vector [37]. It is also important to note that binning is unstable so it should be used with caution in the algorithm. There are numerous ways to transform persistence diagrams for machine learning like persistence landscapes [38] and persistence images [39]. The last step is to combine the topological features with machine learning algorithms. Essentially, these features can be used directly in supervised learning models. Depending on the learning models, we should consider different feature selection and representation models to bring out the best performance of the model. For detailed explanation check article [40].

### Supervised machine learning algorithms

The goal of supervised learning is to build a concise model of the distribution of class labels in terms of predictor features. The resulting classifier is then used to assign class labels to the testing samples where the values of the predictor feature are known, but the value of the class labels are unknown. The classifier’s evaluation is most often based on prediction accuracy (the percentage of correct prediction divided by the total number of predictions) [41]. In this application, we have chosen Decision Trees classifiers as logic learning methods, and Linear Discriminant Analysis, Random Forest Classifier, Support Vector Machines and Naïve Bayes Classification as statistical learning algorithms.

*Decision Tree Classifier (DT)* A decision tree classifier is a non-parametric classifier that does not require any a priori statistical assumptions to be made regarding the distribution of data. The basic structure of the decision tree, however, consists of one root node, a number of internal nodes and finally a set of terminal nodes. A node is a subset of the predictors that is used to determine a split. A non-terminal node or parent node is a node that is further split into two child nodes. Growing a tree consists of selecting the optimal splits to determine a non-terminal node, and the assignment of each terminal node to a class. The data is recursively divided down the decision tree according to the defined classification framework.

*Multi-Class Support Vector Machine (SVM)* The support vector machines (SVMs) are a set of related learning algorithms used for classification and regression. Like the Decision Tree classifiers, the SVM are non-parametric classifiers. The most basic scheme used for the implementation of SVM multi-class classification is the one-against-all method. In this simplest extension of the SVM to a K-class problem, K binary SVM models are constructed. In ${\text{k}}^{{{\text{th}}}}$ class SVM problem, class ${\text{c}}_{{\text{k}}}$ is separated from the remaining classes. All k binary SVM classifiers are combined together to make a final multi-class classifier. Here the remaining means that all the data points from classes other than ${\text{c}}_{{\text{k}}}$ are combined to form one class ${\text{c}}_{{\text{l}}}$. The optimal hyperplane that separates data points from the class ${\text{c}}_{{\text{i}}}$ and the combined class ${\text{c}}_{{\text{l}}}$ is found by using the standard SVM approach.

*Naive Bayesian classifier (NB)* The naive Bayesian classifier is simple probabilistic classifier based on Bayes’ theorem with independence assumptions between predictors. A naive Bayesian model is easy to build, with no complicated iterative parameter estimation which makes its short computational time for training. The parameter of naive Bayes models estimates by the method of maximum likelihood. The Naive Bayes classifier reduce the intractable sample complexity by making a conditional independence assumption that dramatically reduces the number of parameters to be estimated when modeling P(X|Y).

*Linear Discriminant Analysis (LDA)* LDA is a common technique used for dimensionality reduction and classification that has been extensively used for processing data information for machine learning and pattern classification applications. The LDA technique is developed to transform the features into a lower dimensional space, which maximizes the ratio of the between-class variance to the within-class variance, thereby guaranteeing maximum class separability.

*Random Forest Classifier (RF)* A random forest is a classifier consisting of a collection of tree-structured classifiers {*h*(**x**, $\theta_{k}$), *k* = 1,…} where the {$\theta_{k}$} are independent identically distributed random vectors and each tree casts a unit vote for the most popular class at input **x**. An upper bound for random forests can be derived for the generalization error in terms of two parameters that are measures of how accurate the individual classifiers are and of the dependence between them. The interplay between these two gives the foundation for understanding the workings of random forests.

### Proposed methodology

**Fig. 5:**
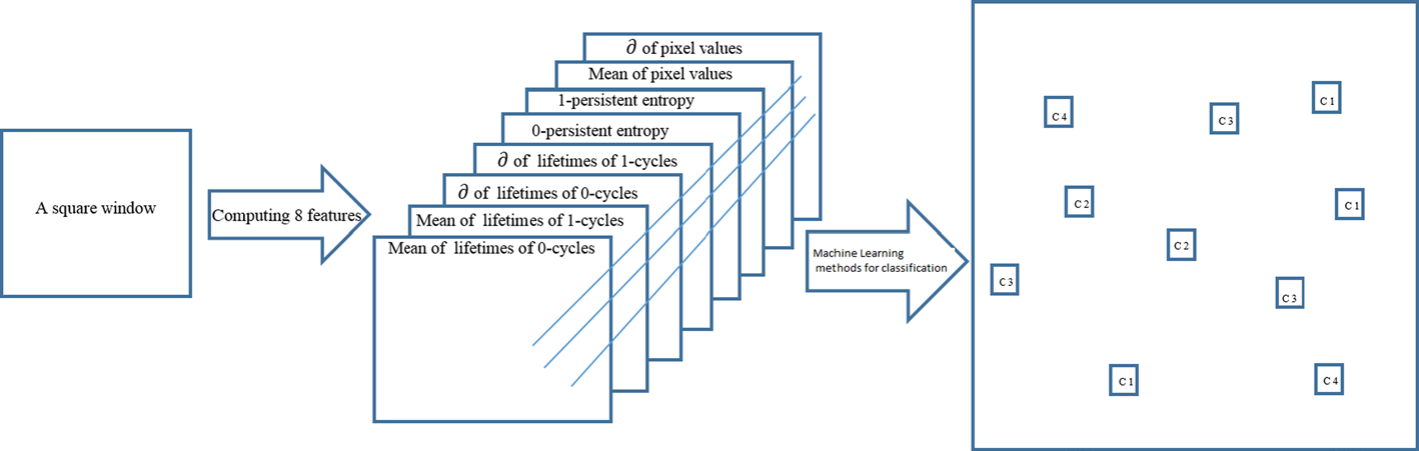
Computation of 8 features inside each sliding window to get the classes using machine learning methods

The topological features calculated using persistent homology have a big importance in image segmentation [42–44]. We compute topological features over windows in glands. Machine Learning techniques are then used to predict the classes of each window, which allow us to know the class of each Gland. Then we calculate the precision metric over all the Gland in each image. We apply the persistence algorithm to the image, exactly to the derived functions given by the pixel values. More explicitly, we manipulate images by an overlapping square sliding window of size 50 * 50. We applied our methodology on 40 * 40 and 60 * 60 window sizes (showed in the Additional file 1 at the end of the article). Changing the window size to 40 or 60 has a small effect on performance and slight differences in classification results are observed. After computation of persistent homology in each window we can get the life duration of 0-cycles and of 1-cycles as well as the persistent entropy. Besides topological features, we calculate the mean and the standard deviation of the life durations of 0-cycles and 1-cycles in each window, their persistent entropies for dimensions 0 and 1, and the mean and standard deviation of pixel values, which will form a set of eight features calculated in each patch. The persistent entropy H of the topological space is calculated as follows:

$$H = - \mathop \sum \limits_{i \in I} p_{i} \log \left( {p_{i} } \right)$$

where $i = \frac{{l_{i} }}{L}$, $l_{i} = b_{i} - a_{i}$ and $L = \mathop \sum \limits_{i \in I} l_{i}$. Note that, when topological noise is present, for each dimension of the Betti barcode, there can be more than one line, denoted by [$a_{i} ;\infty$], with $i \in I$. Instead of $\left[ {a_{i} ; \infty } \right)$ a persistent topological feature is denoted by $\left[ {a_{i} ; m} \right)$ where $m = \max \left\{ F \right\} + 1$. Note that the maximum persistent entropy corresponds to the situation in which all of the lines in the barcode are of equal length. Many articles described the use of these statistical features derived from persistent homology especially persistent entropy [45–47]. These methods are mathematically robust methods of turning a persistence diagram into a stable feature vector. The Fig. 5, shows how we get a vector of 8 features from each sliding window then we apply machine learning methods to classify each image.

In this study we intend to study the Gleason score on some glands issued from a new optical microscopy technique called SLIM. The Spatial Light Interference Microscopy (SLIM) is a new optical microscopy technique that combines two classic ideas in light imaging: Zernike’s phase contrast microscopy and Gabor’s holography. SLIM provides quantitative phase images of transparent (unstained) structures with 0.3 nm spatial accuracy. This technique reveals the intrinsic contrast of cell structures and, in addition, renders quantitative optical path-length maps across tissues. First, we break down each prostate cancer images issued from the SLIM technique by an image processing program called “ImageJ” into K-glands. Then the topological features specifically, persistence homological features, are extracted in sliding windows inside each gland. Methods issued from machine learning to classify these windows into their corresponding Gleason score are applied. Note that the machine learning techniques applied on homological persistence features are very effective in the detection of the right Gleason score of the prostate cancer in these kinds of images. Finally, we compute the confusion matrix to measure the accuracy of the five supervised machine learning methods used.

## Results and application

### Data collection

**Fig. 6:**
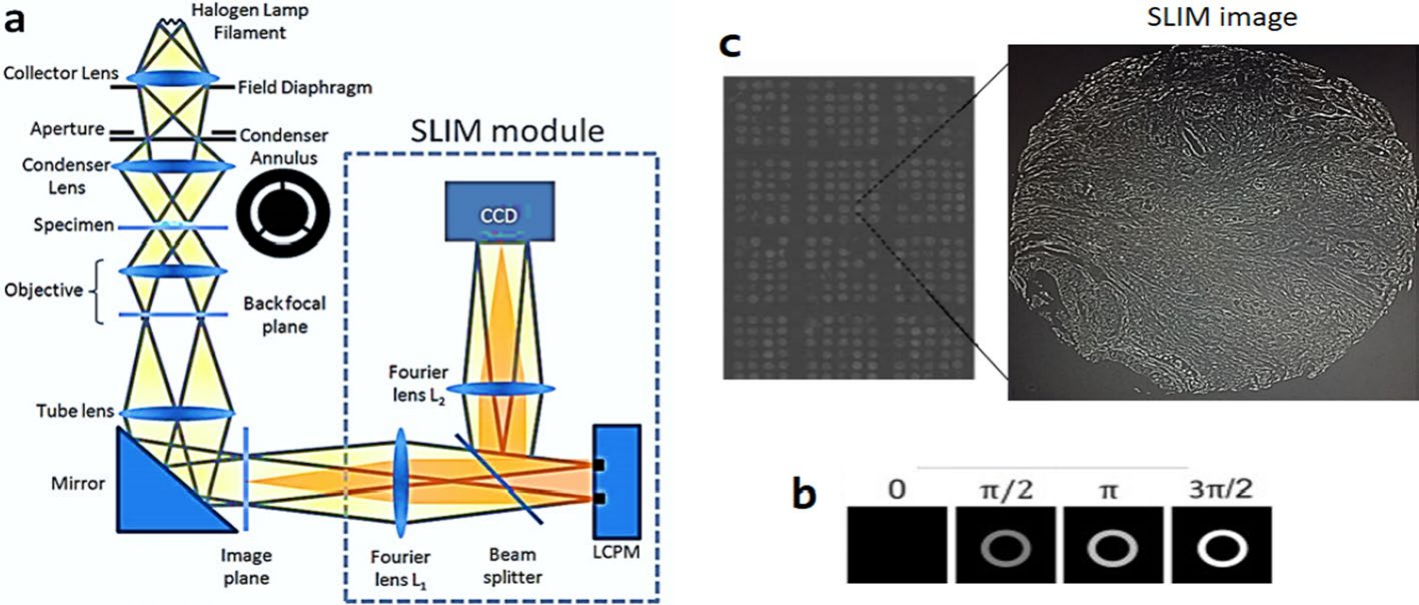
**a**: The SLIM module added on to a commercial phase contrast microscope. **b**: Four frames are acquired to compute one phase image by modulating the phase difference between scattered and incident light using a spatial light modulator (SLM). **c**:An image of the whole slide scanned using SLIM and an esemble of gleason pattern through SLIM image

The Quantitative Light Imaging (QLI) group at the Beckman Institute for Advanced Science and Technology at the University of Illinois at Urbana-Champaign (UIUC) recently developed a new technology called Spatial Light Interference Microscopy [48]. SLIM is an add-on module for an existing phase contrast microscope that has the potential to have a big impact on the area of light microscopy. We introduce SLIM as a unique, extremely sensitive QPI method that has the potential to enable unparalleled structural and dynamics studies in biology and beyond. Zernike's phase contrast approach, which reveals the intrinsic contrast of transparent samples [49], is combined with Gabor's holography, which renders quantitative phase maps throughout the sample [50]. SLIM enables speckle-free imaging with sub-nanometer spatial background noise because to the illuminating light's exceptionally short coherence length of around 1.2 μm. Further, the SLIM images are also intrinsically registered with the microscope's other channels, including fluorescence, allowing for strong multimodal investigations. The results proved that SLIM could acquire rich and quantitative information from biological structures without physical contact or staining (see Fig. 6). The following are the primary characteristics of SLIM. Speckle-free pictures are provided by SLIM, allowing for spatially sensitive optical path-length assessment (0.3 nm). It uses common path interferometry to quantify optical route length in a temporally sensitive manner (0.03 nm).

Overtime researches has shown that statistical analysis of SLIM images can identify and locate tumor areas in high degree of malignancy prostate cancer biopsies. The information relevant to the diagnosis, especially for low-malignancy biopsies, is drowned in the large body of data from the SLIM microscopy, which provides images in gray levels of about 50,000 × 50,000 pixels for a 40 × resolution acquisition on a tissue cut of about 1 cm^2^. It is therefore necessary to make an adequate treatment to extract and present it in intelligible form. In addition, the amount of data in a SLIM image is substantial and it becomes necessary to integrate it to extract the information. This integration requires a shift from the local to the global as proposed by the algebraic topology. Our approach concentrates on the theoretical and fundamentally on the practical development of new algebraic topological tools and mainly persistence homology.

Noting that in our application, we have a data set of 500 images (of size 10,000 * 10,000) approximately, classified into 5 classes. Each image is decomposed of glands of size 1000 * 1000 in average. All the glands in one image are of the same class. Then we compute 8 scores on sliding windows of size 50 * 50 on these glands. The machine learning methods are applied after that on these windows to classify them. As an example, if we have one image that consist of 50 glands, and each gland consists of 20 windows. So, we will get 1000 windows for the image. We run the machine learning methods to predict the classes of each window and then we can check the most common class in each gland. After that we can calculate the accuracy for each image. And finally, we calculate the global accuracy for all images.

### Statistical analysis

A confusion matrix is displayed to describe the performance of the classifiers and measure the accuracy of them. Aside from confusion matrix, cross-validation is primarily used in applied machine learning to estimate the skill of a machine learning model on unseen data. That is, to use a limited sample in order to estimate how the model is expected to perform in general when used to make predictions on data not used during the training of the model. It is a popular method because it is simple to understand and because it generally results in a less biased or less optimistic estimate of the model skill than other methods, such as a simple train/test split. We hired in our study five classification algorithms: the decision tree (DTC), Random Forest (RF), support vector machine classifier (SVM), Naïve Bayes classifier (NBC) and linear discriminant analysis classifier (LDA).

Cross-validation is a model assessment technique used to evaluate a supervised classification algorithm’s performance in making predictions on new datasets that it has not been trained on. This is done by partitioning a dataset and using a subset to train the supervised learning algorithms and the remaining data for validation and testing. Several methods of cross validation exist such as k-fold, Holdout, Leave-out and Re-substitution. We use k-fold method that partitions the persistent homology features of glands images into k randomly chosen subsets of roughly equal size. One subset is used to validate the model, one subset is used to testing the model, training the model using the remaining subsets. This process is repeated T = 100 sampling such that each subset is used exactly once for validation. A ROC (Receiver Operating Characteristic) curve summarizes the performance of a classifier over all possible thresholds. It is generated by plotting the True Positive Rate (y-axis) against the False Positive Rate (x-axis) by varying the threshold for assigning observations to a given class. ROC curves are used to compare different supervised classifiers. If the curve has high values, it leads to the greater of area under the curve obtained (AUC), and the less error the classifier makes.

*Gleason score 3* According to Gleason grade system, grade 3 is characterized by small glands, thin boundary, small and round lumen. This score has three intra classes labeled as class 32, class 33, and class 34. We used the same k-fold cross validation with k = 2, 3, 4, 5 on the intra classes to measure the accuracy of each of the five classifiers individually. Table 1 shows the performance of our methodology combining persistent homology to extract features from images and supervised algorithms to classify the Gleason score 3 of prostate cancer images by calculate the accuracy of confusion matrix and the area under the roc curve (AUC). The confusion matrix was implemented, and the accuracy was comprised as 97.4% for the decision tree classifier, 65.7% for the support vector machine, 65.9% for the random forest classifier, 56.7% for the Naïve Bayes classifier, and 55.1% for the linear discriminant analysis classifier. The greater of area under the curve obtained (AUC) for the decision tree classifier with 0.9813 value of k = 3 fold CV.

**Table 1:** Results of persistent homology application combined with supervised algorithms with different k-fold cross-validation value for the classification of Gleason score 3 of cancerous prostate gland images

k-fold/method	LDA		NBC		SVM		DTC		RF	
	Accuracy (%)	AUC	Accuracy (%)	AUC	Accuracy (%)	AUC	Accuracy (%)	AUC	Accuracy (%)	AUC
K = 2 fold ($${\raise0.7ex\hbox{$1$} \!\mathord{\left/ {\vphantom {1 2}}\right.\kern-\nulldelimiterspace} \!\lower0.7ex\hbox{$2$}}$$ training $${\raise0.7ex\hbox{$1$} \!\mathord{\left/ {\vphantom {1 2}}\right.\kern-\nulldelimiterspace} \!\lower0.7ex\hbox{$2$}}$$ testing)	53.7	0.5042	52.4	0.5523	63.5	0.7378	**95.2**	**0.9652**	65.6	0.7830
K = 3 fold ($${\raise0.7ex\hbox{$2$} \!\mathord{\left/ {\vphantom {2 3}}\right.\kern-\nulldelimiterspace} \!\lower0.7ex\hbox{$3$}}$$ training $${\raise0.7ex\hbox{$1$} \!\mathord{\left/ {\vphantom {1 3}}\right.\kern-\nulldelimiterspace} \!\lower0.7ex\hbox{$3$}}$$ testing)	54.6	0.5065	53.5	0.5762	64.2	0.7422	**97.4**	**0.9813**	65.7	0.7831
K = 4 fold ($${\raise0.7ex\hbox{$3$} \!\mathord{\left/ {\vphantom {3 4}}\right.\kern-\nulldelimiterspace} \!\lower0.7ex\hbox{$4$}}$$ training $${\raise0.7ex\hbox{$1$} \!\mathord{\left/ {\vphantom {1 4}}\right.\kern-\nulldelimiterspace} \!\lower0.7ex\hbox{$4$}}$$ testing)	53.5	0.5035	54.8	0.5728	64.8	0.7431	**97.2**	**0.9789**	65.8	0.7855
K = 5 fold ($${\raise0.7ex\hbox{$4$} \!\mathord{\left/ {\vphantom {4 5}}\right.\kern-\nulldelimiterspace} \!\lower0.7ex\hbox{$5$}}$$ training $${\raise0.7ex\hbox{$1$} \!\mathord{\left/ {\vphantom {1 5}}\right.\kern-\nulldelimiterspace} \!\lower0.7ex\hbox{$5$}}$$ testing)	55.1	0.5135	56.7	0.5823	65.7	0.7586	**96.9**	**0.9755**	65.9	0.7863

The bold signifies the start and the end of the procedure of classification

*Gleason score 4* According to Gleason grade system, the glands in grade 4 merge together and create a mass of glands containing multiple lumens. This score has three intra classes labeled as class 43, class 44, and class 45. We used the same k-fold cross validation with k = 2, 3, 4, 5 on the intra classes to measure the precision of each of the five classifiers individually. Table 2 shows the performance of our methodology combining persistent homology to extract features from images and supervised algorithms to classify the Gleason score 4 of prostate cancer images by calculate the accuracy of confusion matrix and the area under the roc curve (AUC).The confusion matrix was displayed, and the accuracy was comprised as 97.8% for the decision tree classifier, 77.8% for the random forest classifier, 67.5% for the support vector machine, 44.8% for the Naïve Bayes classifier, and 46.3% for the linear discriminant analysis classifier. To quantify the results, we calculate the area under the roc curve (AUC) for each supervised method with k = 2-, 3-, 4- and 5-fold CV. We obtained the decision tree classifier giving the highest value of the AUC compared to the other methods.

**Table 2:** Results of persistent homology application combined with supervised algorithms with different k-fold cross-validation value for the classification of Gleason score 4 of cancerous prostate gland images

k-fold/method	LDA		NBC		SVM		DTC		RF	
	Accuracy (%)	AUC	Accuracy (%)	AUC	Accuracy (%)	AUC	Accuracy (%)	AUC	Accuracy (%)	AUC
K = 2 fold ($${\raise0.7ex\hbox{$1$} \!\mathord{\left/ {\vphantom {1 2}}\right.\kern-\nulldelimiterspace} \!\lower0.7ex\hbox{$2$}}$$ training $${\raise0.7ex\hbox{$1$} \!\mathord{\left/ {\vphantom {1 2}}\right.\kern-\nulldelimiterspace} \!\lower0.7ex\hbox{$2$}}$$ testing)	43.4	0.4781	42.4	0.4346	61.6	0.8117	**97.8**	**0.9871**	76.6	0.8445
K = 3 fold ($${\raise0.7ex\hbox{$2$} \!\mathord{\left/ {\vphantom {2 3}}\right.\kern-\nulldelimiterspace} \!\lower0.7ex\hbox{$3$}}$$ training $${\raise0.7ex\hbox{$1$} \!\mathord{\left/ {\vphantom {1 3}}\right.\kern-\nulldelimiterspace} \!\lower0.7ex\hbox{$3$}}$$ testing)	43.6	0.4782	42.8	0.4410	63.4	0.8152	**97. 4**	**0.9821**	77.8	0.8493
K = 4 fold ($${\raise0.7ex\hbox{$3$} \!\mathord{\left/ {\vphantom {3 4}}\right.\kern-\nulldelimiterspace} \!\lower0.7ex\hbox{$4$}}$$ training $${\raise0.7ex\hbox{$1$} \!\mathord{\left/ {\vphantom {1 4}}\right.\kern-\nulldelimiterspace} \!\lower0.7ex\hbox{$4$}}$$ testing)	45.1	0.4884	43.5	0.4431	67.1	0.8233	**97.9**	**0.9867**	76.8	0.8353
K = 5 fold ($${\raise0.7ex\hbox{$4$} \!\mathord{\left/ {\vphantom {4 5}}\right.\kern-\nulldelimiterspace} \!\lower0.7ex\hbox{$5$}}$$ training $${\raise0.7ex\hbox{$1$} \!\mathord{\left/ {\vphantom {1 5}}\right.\kern-\nulldelimiterspace} \!\lower0.7ex\hbox{$5$}}$$ testing)	46.3	0.4921	44.8	0.4780	67.5	0.8291	**97.1**	**0.9819**	76.5	0.8348

The bold signifies the start and the end of the procedure of classification

*Gleason score 5* According to Gleason grade system, grade 5 indicates that poorly differentiated tissue and low chance of survival. This score has two intra classes labeled as class 53 and class 55. We also used the same k-fold cross validation with k = 2, 3, 4, 5 on the intra classes to measure the fineness of each of the five classifiers individually. Table 3 shows the performance of our methodology combining persistent homology to extract features from images and supervised algorithms to classify the Gleason 5 score of prostate cancer images. The accuracy of confusion matrix was executed, and the accuracy is understood as 99.3% for the decision tree classifier, 80.1% for the random forest classifier, 84.5% for the support vector machine, 90.8% for the Naïve Bayes classifier, and 73.2% for the linear discriminant analysis classifier. To quantify the results, we calculate the area under the roc curve (AUC) for each supervised method with k = 2-, 3-, 4- and 5-fold CV, we obtained the decision tree classifier giving the highest value of the AUC compared to the other methods.

**Table 3:** Results of persistent homology application combined with supervised algorithms with different k-fold cross-validation value for the classification of the Gleason score 5 of cancerous prostate gland images

k-fold/method	LDA		NBC		SVM		DTC		RF	
	Accuracy (%)	AUC	Accuracy (%)	AUC	Accuracy (%)	AUC	Accuracy (%)	AUC	Accuracy (%)	AUC
K = 2 fold ($${\raise0.7ex\hbox{$1$} \!\mathord{\left/ {\vphantom {1 2}}\right.\kern-\nulldelimiterspace} \!\lower0.7ex\hbox{$2$}}$$ training $${\raise0.7ex\hbox{$1$} \!\mathord{\left/ {\vphantom {1 2}}\right.\kern-\nulldelimiterspace} \!\lower0.7ex\hbox{$2$}}$$ testing)	71.7	0.8354	87.7	0.8859	82.2	0.8326	**99.2**	**0.9941**	79.4	0.8560
K = 3 fold ($${\raise0.7ex\hbox{$2$} \!\mathord{\left/ {\vphantom {2 3}}\right.\kern-\nulldelimiterspace} \!\lower0.7ex\hbox{$3$}}$$ training $${\raise0.7ex\hbox{$1$} \!\mathord{\left/ {\vphantom {1 3}}\right.\kern-\nulldelimiterspace} \!\lower0.7ex\hbox{$3$}}$$ testing)	73.1	0.8407	88.8	0.9106	84.4	0.8546	**99.4**	**0.9959**	79.1	0.8547
K = 4 fold ($${\raise0.7ex\hbox{$3$} \!\mathord{\left/ {\vphantom {3 4}}\right.\kern-\nulldelimiterspace} \!\lower0.7ex\hbox{$4$}}$$ training $${\raise0.7ex\hbox{$1$} \!\mathord{\left/ {\vphantom {1 4}}\right.\kern-\nulldelimiterspace} \!\lower0.7ex\hbox{$4$}}$$ testing)	73.2	0.8407	89.4	0.9284	85.1	0.8566	**98.9**	**0.9893**	80.1	0.8586
K = 5 fold ($${\raise0.7ex\hbox{$4$} \!\mathord{\left/ {\vphantom {4 5}}\right.\kern-\nulldelimiterspace} \!\lower0.7ex\hbox{$5$}}$$ training $${\raise0.7ex\hbox{$1$} \!\mathord{\left/ {\vphantom {1 5}}\right.\kern-\nulldelimiterspace} \!\lower0.7ex\hbox{$5$}}$$ testing)	71.9	0.8361	90.8	0.9355	84.5	0.8356	**99.3**	**0.9889**	79.4	0.8558

The bold signifies the start and the end of the procedure of classification

### Discussion

Depending on the approached outcomes, there is a clear evidence that the decision tree is the most preferable supervised machine learning algorithm among the other classifiers. It has shown an accuracy above 95% of the classification. Consequently, it is the bestead classifier who was able to detect the accurate diagnosis through the intra classes of Gleason score 3, Gleason score 4, and Gleason score 5 solely. Besides it was considered to be the leading classifier of our study. Our destination then focused on the tabulation of the decision tree when taking all Gleason scores jointly. Here we picked several images from each of the four Gleason scores including Gleason score 2 that has one single class that is class 23. In other embodiments, we stratified this classifier on Gleason score 2, Gleason score 3, Gleason score 4, and Gleason score 5 altogether but with different k-fold cross validation. Cross-Validation (CV) is recognized as a very powerful tool. It helps better use the data and gives us much more information about our algorithm performance. K-fold cross validation is about estimating the accuracy, not improving the accuracy where most implementations of k-fold cross validation give an estimate of how accurately they are measuring the accuracy. Table 4 shows the performance of our methodology combining persistent homology to extract features from images and supervised algorithms to classify the Gleason score 2, Gleason score 3, Gleason score 4, and Gleason score 5 of prostate cancer images by calculate the accuracy of confusion matrix and the area under the roc curve (AUC). The results were implicated as 95.8% for 2-fold CV, 95.4% for 3-fold CV, 96.1% for 4-fold CV, and 96.9% for 5-fold CV. As a result, the fluctuation of k-fold cross validation did not affect the fineness of the results. Hence cross validation was considered posteriorly as an inconsequential procedure in our case. The greater of area under the roc curve obtained (AUC) for the decision tree classifier with k = 2-, 3-, 4- and 5-fold CV. Results on 40 * 40 and 60 * 60 window sizes are added as Additional file 1 at the end of the article. Small effect on performance were remarked with slight differences in classification results.

**Table 4:** Results of persistent homology application combined with supervised algorithms with different k-fold cross-validation value for discrimination of the Gleason score 2, 3, 4 and 5 of cancerous prostate gland images

k-fold/method	LDA		NBC		SVM		DTC		RF	
	Accuracy (%)	AUC	Accuracy (%)	AUC	Accuracy (%)	AUC	Accuracy (%)	AUC	Accuracy (%)	AUC
K = 2 fold ($${\raise0.7ex\hbox{$1$} \!\mathord{\left/ {\vphantom {1 2}}\right.\kern-\nulldelimiterspace} \!\lower0.7ex\hbox{$2$}}$$ training $${\raise0.7ex\hbox{$1$} \!\mathord{\left/ {\vphantom {1 2}}\right.\kern-\nulldelimiterspace} \!\lower0.7ex\hbox{$2$}}$$ testing)	48.5	0.5671	50.5	0.5671	56.7	0.6162	**95.8**	**0.9889**	66.5	0.8242
K = 3 fold ($${\raise0.7ex\hbox{$2$} \!\mathord{\left/ {\vphantom {2 3}}\right.\kern-\nulldelimiterspace} \!\lower0.7ex\hbox{$3$}}$$ training $${\raise0.7ex\hbox{$1$} \!\mathord{\left/ {\vphantom {1 3}}\right.\kern-\nulldelimiterspace} \!\lower0.7ex\hbox{$3$}}$$ testing)	50.2	0.5722	52.3	0.5822	55.9	0.6233	**95.4**	**0.9896**	67.8	0.8221
K = 4 fold ($${\raise0.7ex\hbox{$3$} \!\mathord{\left/ {\vphantom {3 4}}\right.\kern-\nulldelimiterspace} \!\lower0.7ex\hbox{$4$}}$$ training $${\raise0.7ex\hbox{$1$} \!\mathord{\left/ {\vphantom {1 4}}\right.\kern-\nulldelimiterspace} \!\lower0.7ex\hbox{$4$}}$$ testing)	51.2	0.5971	51.7	0.5945	58.2	0.6541	**96.1**	**0.9879**	66.7	0.8202
K = 5 fold ($${\raise0.7ex\hbox{$4$} \!\mathord{\left/ {\vphantom {4 5}}\right.\kern-\nulldelimiterspace} \!\lower0.7ex\hbox{$5$}}$$ training $${\raise0.7ex\hbox{$1$} \!\mathord{\left/ {\vphantom {1 5}}\right.\kern-\nulldelimiterspace} \!\lower0.7ex\hbox{$5$}}$$ testing)	53.6	0.6149	53.6	0.6156	59.6	0.6728	**96.9**	**0.9929**	68.0	0.8237

The bold signifies the start and the end of the procedure of classification

On the other hand, deep learning methods were applied to classify the images. A CNN method showed a 72% accuracy of classification of the 4 classes of Gleason score (2, 3, 4, 5). For the subclasses (as 2–2, 2–3…), also the CNN was not a good classifier and our method remains the only one with a high accuracy of classification of Gleason score subclasses.

### Existing methods

Overtime there was various proposals discussing the importance of persistent homology and others examining the renovation of machine learning in the medical field. Yet, few tutored the integration of these two on image segmentation for Gleason score stratification. Presently we focus in this study on two existing methods and compare their results to ours. The authors of the article “Quantitative Phase Imaging” [51] proposed a unified framework to perform automatic diagnosis of prostatic cancer directly from QPI images. The framework used texture analysis to produce segmentation maps for different regions of the cores. Given the label map, all glands in each core are identified and their feature vectors are calculated. Bag-of-Ward (BoW) model was used to compute the core feature vector from its glands. The main two common points were the proposition to study the Gleason score on glands issued from the SLIM optical microscopy technique and the employment of the SVM classifier. Researchers trained the random forest classifier to segment SLIM based on the texture of the tissue and SVM classifier to differentiate Gleason grade 3 and 4. In addition, PCA and K-means clustering were employed as well. Eventually, using SVM classifiers, the accuracy was 70% in the binary classification problem for grade 4 and 3. Thus the preference in our work is the manifest of SVM and four more supervised classifiers to detect the right Gleason score not only for grades 3 and 4 but also between the intra classes of these grades. Besides, we were able to recognize a better precision with accuracy exceeded 90%.

On the other hands, other authors in their study “Persistent Homology for the Quantitative Evaluation of Architectural Features in Prostate Cancer Histology” [19] proposed persistent homology as a new means of describing prostate cancer architecture (PCa). The rapprochement between both studies is the integration of machine learning with persistent homology. First they applied persistent homology to prostate cancer glandular architecture, they computed topological representations of purely graded prostate cancer histopathology images of Gleason patterns 3, 4 and 5, and showed that persistent homology was capable of clustering prostate cancer histology into architectural groups through a ranked persistence vector. Then un-supervised learning techniques, mainly principal component analysis (PCA) and Hierarchical Clustering, were executed. The results of their study displayed the ability to stratify PCa into architectural subtypes using persistent homology as a tool, which ultimately might have different prognostic outcomes. Of particular interest was the ability to segregate PCa architecture into potentially prognostically distinct groups. Whereas the advantage in our work is the deployment of five supervised machine learning algorithms: decision tree classifier, SVM classifier, Naïve Bayes classifier, and linear discriminant analysis on the extracted persistent homological features. Moreover, we utilized these classification models on Gleason score 2, Gleason score 3, Gleason score 4, Gleason score 5. Consequently, and due to our outcomes, decision tree classifier was superb classifier among the five classifiers who was able to differentiate the scores and the intra classes of each Gleason score. Substantially, such results might help pathologists better comprehend the Gleason grading system to snaffle subservient clinical information.

## Conclusion

Many works on images have employed persistent homology and associated statistical conclusions to classify the data. We employed the statistical results of persistent homology to classify photos into their Gleason scores in our research. To compute persistent homology, the pixels are binarized based on a threshold. The Gleason score reveals a revolutionary tumor detection technique, which is provided utilizing the novel concept of persistent homology profiles. In Gleason disclosure, combining machine learning with persistent homology is recommended as an effective technique. The topological features extracted from biomedical pictures, notably the SLIM technology, are represented as the scale and distribution of zero and one-dimensional persistence features via persistent homology. We present a unified platform for doing automatic prostate cancer diagnosis. The framework uses texture analysis to create segmentation maps for various gland regions. Given the label map, all glands are identified and their topological features are calculated. ImageJ is used to pluck the features from the glands and five distinct standard classifiers were used then. The decision tree classifier was the leading classifier among the rest and showed an accuracy of above 90% obtained in the binary classification problem for Gleason scores 2, 3, 4 and 5 jointly. Moreover, this classifier also performed overwhelming, above 90% fineness, results in the binary classification for intra classes in Gleason score 3, Gleason score 4, and Gleason score 5 individually.

## Future work

While this is a preliminary work and yields no definitive conclusions regarding clinical utility, it does point to the power of persistent homology based on machine learning, PHML, to interrogate the architectures present in PCa. Using this tool, it may be possible to develop a better understanding of not only inter and intra classes of Gleason score in PCa but, more importantly, how they correlate with patient prognostic outcomes. There are many directions for further research. On the processing front, it is serious to examine the effectively of our results to characterize the aggressiveness of prostate cancer and allow urologists to make more informed decisions. On the technology front, it is useful to study the applicability of our approach to others by using convolutional neural network (CNN) to extract the features instead of PH.

## Supplementary Information

**Additional file 1**

## Data Availability

All data, models, and code generated or used during the study appear in the submitted article and are provided upon request by contacting Abbas Rammal via email: rammal_abbass@hotmail.com.
