# Supplementary material for: Machine learning techniques on homological persistence features for prostate cancer diagnosis
Source: BMC Bioinformatics. 2022 Nov 12;23:476. doi: 10.1186/s12859-022-04992-5 (PMC9652917; doi:10.1186/s12859-022-04992-5)
Supplement: Supplementary file 1 — Additional file 1 [file 12859_2022_4992_MOESM1_ESM.docx]

**_Supplementary Material:_**

**Results of 40*40 windows sizes:**

Table 1. Results of persistent homology application combined with supervised algorithms with different k-fold cross-validation value for the classification of Gleason score 3 of cancerous prostate gland images.

| k-fold / Method | **LDA** |  | **NBC** |  | **SVM** |  | **DTC** |  | **RF** |  |
| --- | --- | --- | --- | --- | --- | --- | --- | --- | --- | --- |
|  | Accuracy | AUC | Accuracy | AUC | Accuracy | AUC | Accuracy | AUC | Accuracy | AUC |
| K = 2 fold  ($\frac{1}{2}$ training $\frac{1}{2}$ testing) | 51.2 % | 0.5011 | 50.4 % | 0.5413 | 59.8 % | 0.6884 | **93.7 %** | **0.9551** | 62.6 % | 0.7402 |
| K = 3 fold  ($\frac{2}{3}$ training $\frac{1}{3}$ testing) | 52.9 % | 0.5121 | 50.7 % | 0.5591 | 63.8 % | 0.7204 | **95.8 %** | **0.9788** | 63.5 % | 0.7715 |
| K = 4 fold  ($\frac{3}{4}$ training $\frac{1}{4}$ testing) | 53.2 % | 0.5031 | 53.9 % | 0.5735 | 65.2 % | 0.7350 | **96.9 %** | **0.9779** | 63.5 % | 0.7685 |
| K = 5 fold  ($\frac{4}{5}$ training $\frac{1}{5}$ testing) | 54.7 % | 0.5112 | 54.9 % | 0.5563 | 63.8 % | 0.7267 | **96.8%** | **0.9695** | 64.1 % | 0.7754 |

Table 2. Results of persistent homology application combined with supervised algorithms with different k-fold cross-validation value for the classification of Gleason score 4 of cancerous prostate gland images.

| k-fold / Method | **LDA** |  | **NBC** |  | **SVM** |  | **DTC** |  | **RF** |  |
| --- | --- | --- | --- | --- | --- | --- | --- | --- | --- | --- |
|  | Accuracy | AUC | Accuracy | AUC | Accuracy | AUC | Accuracy | AUC | Accuracy | AUC |
| K = 2 fold  ($\frac{1}{2}$ training $\frac{1}{2}$ testing) | 45.7 % | 0.4821 | 44.1 % | 0.4452 | 63.2 % | 0.7720 | **97.5 %** | **0.9792** | 78.5 % | 0.8521 |
| K = 3 fold  ($\frac{2}{3}$ training $\frac{1}{3}$ testing) | 44.8 % | 0.4825 | 44.2 % | 0.4524 | 65.6 % | 0.7922 | **97.6 %** | **0.9805** | 79.6 % | 0.8641 |
| K = 4 fold  ($\frac{3}{4}$ training $\frac{1}{4}$ testing) | 46.4 % | 0.4943 | 47.5 % | 0.4672 | 66.8 % | 0.7957 | **98.1 %** | **0.9852** | 76.8 % | 0.8504 |
| K = 5 fold  ($\frac{4}{5}$ training $\frac{1}{5}$ testing) | 48.7 % | 0.5064 | 48.8 % | 0.4733 | 65.5 % | 0.8012 | **97.5 %** | **0.9825** | 75.5 % | 0.8248 |

Table 3. Results of persistent homology application combined with supervised algorithms with different k-fold cross-validation value for the classification of the Gleason score 5 of cancerous prostate gland images.

| k-fold / Method | **LDA** |  | **NBC** |  | **SVM** |  | **DTC** |  | **RF** |  |
| --- | --- | --- | --- | --- | --- | --- | --- | --- | --- | --- |
|  | Accuracy | AUC | Accuracy | AUC | Accuracy | AUC | Accuracy | AUC | Accuracy | AUC |
| K = 2 fold  ($\frac{1}{2}$ training $\frac{1}{2}$ testing) | 73.8 % | 0.8265 | 86.8 % | 0.8630 | 83.5 % | 0.8246 | **98.4 %** | **0.9894** | 82.3 % | 0.8477 |
| K = 3 fold  ($\frac{2}{3}$ training $\frac{1}{3}$ testing) | 74.5 % | 0.8355 | 87.1 % | 0.8835 | 85.3 % | 0.8462 | **98.8 %** | **0.9923** | 81.9 % | 0.8475 |
| K = 4 fold  ($\frac{3}{4}$ training $\frac{1}{4}$ testing) | 73.9 % | 0.8240 | 88.7 % | 0.9001 | 85.4 % | 0.8378 | **97.5 %** | **0.9873** | 85.1 % | 0.8532 |
| K = 5 fold  ($\frac{4}{5}$ training $\frac{1}{5}$ testing) | 72.5 % | 0.8338 | 89.2 % | 0.9084 | 82.7 % | 0.8156 | **99.1 %** | **0.9856** | 82.5 % | 0.8586 |

Table 4. Results of persistent homology application combined with supervised algorithms with different k-fold cross-validation value for discrimination of the Gleason score 2, 3, 4 and 5 of cancerous prostate gland images.

| k-fold / Method | **LDA** |  | **NBC** |  | **SVM** |  | **DTC** |  | **RF** |  |
| --- | --- | --- | --- | --- | --- | --- | --- | --- | --- | --- |
|  | Accuracy | AUC | Accuracy | AUC | Accuracy | AUC | Accuracy | AUC | Accuracy | AUC |
| K = 2 fold  ($\frac{1}{2}$ training $\frac{1}{2}$ testing) | 46.9 % | 0.5048 | 48.8 % | 0.5243 | 55.6 % | 0.6088 | **93.5 %** | **0.9534** | 64.5 % | 0.7863 |
| K = 3 fold  ($\frac{2}{3}$ training $\frac{1}{3}$ testing) | 46.5 % | 0.5307 | 50.8 % | 0.5688 | 56.7 % | 0.6165 | **95.4 %** | **0.9759** | 66.9 % | 0.8122 |
| K = 4 fold  ($\frac{3}{4}$ training $\frac{1}{4}$ testing) | 49.8 % | 0.5615 | 52.5 % | 0.5769 | 57.9 % | 0.6285 | **95.8%** | **0.9756** | 67.1 % | 0.8152 |
| K = 5 fold  ($\frac{4}{5}$ training $\frac{1}{5}$ testing) | 53.5 % | 0.5832 | 54.7 % | 0.5838 | 57.6 | 0.6448 | **96.9%** | **0.9877** | 70.8 % | 0.8280 |

**Results of 60*60 windows sizes:**

Table 1. Results of persistent homology application combined with supervised algorithms with different k-fold cross-validation value for the classification of Gleason score 3 of cancerous prostate gland images.

| k-fold / Method | **LDA** |  | **NBC** |  | **SVM** |  | **DTC** |  | **RF** |  |
| --- | --- | --- | --- | --- | --- | --- | --- | --- | --- | --- |
|  | Accuracy | AUC | Accuracy | AUC | Accuracy | AUC | Accuracy | AUC | Accuracy | AUC |
| K = 2 fold  ($\frac{1}{2}$ training $\frac{1}{2}$ testing) | 53.7 % | 0.5042 | 52.4 % | 0.5523 | 63.5 % | 0.7378 | **95.2 %** | **0.9652** | 65.6 % | 0.7830 |
| K = 3 fold  ($\frac{2}{3}$ training $\frac{1}{3}$ testing) | 54.6 % | 0.5065 | 53.5 % | 0.5762 | 64.2 % | 0.7422 | **97.4 %** | **0.9813** | 65.7 % | 0.7831 |
| K = 4 fold  ($\frac{3}{4}$ training $\frac{1}{4}$ testing) | 53.5 % | 0.5035 | 54.8 % | 0.5728 | 64.8 % | 0.7431 | **97.2 %** | **0.9789** | 65.8 % | 0.7855 |
| K = 5 fold  ($\frac{4}{5}$ training $\frac{1}{5}$ testing) | 55.1 % | 0.5135 | 56.7 % | 0.5823 | 65.7 % | 0.7586 | **96.9 %** | **0.9755** | 65.9 % | 0.7863 |

Table 2. Results of persistent homology application combined with supervised algorithms with different k-fold cross-validation value for the classification of Gleason score 4 of cancerous prostate gland images.

| k-fold / Method | **LDA** |  | **NBC** |  | **SVM** |  | **DTC** |  | **RF** |  |
| --- | --- | --- | --- | --- | --- | --- | --- | --- | --- | --- |
|  | Accuracy | AUC | Accuracy | AUC | Accuracy | AUC | Accuracy | AUC | Accuracy | AUC |
| K = 2 fold  ($\frac{1}{2}$ training $\frac{1}{2}$ testing) | 45.1 % | 0.4792 | 44.5 % | 0.4436 | 62.4 % | 0.7940 | **97.5 %** | **0.9643** | 75.4 % | 0.8042 |
| K = 3 fold  ($\frac{2}{3}$ training $\frac{1}{3}$ testing) | 44.8 % | 0.4871 | 44.3 % | 0.4482 | 63.6 % | 0.8055 | **97. 2 %** | **0.9650** | 76.9 % | 0.8055 |
| K = 4 fold  ($\frac{3}{4}$ training $\frac{1}{4}$ testing) | 45.6 % | 0.4982 | 45.1 % | 0.4541 | 66.8 % | 0.8162 | **98.2 %** | **0.9877** | 74.2 % | 0.7982 |
| K = 5 fold  ($\frac{4}{5}$ training $\frac{1}{5}$ testing) | 46.7 % | 0.5084 | 45.7 % | 0.4620 | 66.2 % | 0.8142 | **96.5 %** | **0.9782** | 75.5 % | 0.8160 |

Table 3. Results of persistent homology application combined with supervised algorithms with different k-fold cross-validation value for the classification of the Gleason score 5 of cancerous prostate gland images.

| k-fold / Method | **LDA** |  | **NBC** |  | **SVM** |  | **DTC** |  | **RF** |  |
| --- | --- | --- | --- | --- | --- | --- | --- | --- | --- | --- |
|  | Accuracy | AUC | Accuracy | AUC | Accuracy | AUC | Accuracy | AUC | Accuracy | AUC |
| K = 2 fold  ($\frac{1}{2}$ training $\frac{1}{2}$ testing) | 73.1 % | 0.7882 | 85.1 % | 0.8478 | 83.5 % | 0.8236 | **98.8 %** | **0.9902** | 80.9 % | 0.8280 |
| K = 3 fold  ($\frac{2}{3}$ training $\frac{1}{3}$ testing) | 74.8 % | 0.7922 | 86.2 % | 0.8667 | 85.8 % | 0.8438 | **99.1 %** | **0.9923** | 81.5 % | 0.8342 |
| K = 4 fold  ($\frac{3}{4}$ training $\frac{1}{4}$ testing) | 74.7 % | 0.7987 | 86.7 % | 0.8653 | 86.6 % | 0.8744 | **99.2 %** | **0.9925** | 82.2 % | 0.8651 |
| K = 5 fold  ($\frac{4}{5}$ training $\frac{1}{5}$ testing) | 73.6 % | 0.7845 | 88.5 % | 0.8901 | 85.2 % | 0.8552 | **99.4 %** | **0.9919** | 80.7 % | 0.8678 |

Table 4. Results of persistent homology application combined with supervised algorithms with different k-fold cross-validation value for discrimination of the Gleason score 2, 3, 4 and 5 of cancerous prostate gland images.

| k-fold / Method | **LDA** |  | **NBC** |  | **SVM** |  | **DTC** |  | **RF** |  |
| --- | --- | --- | --- | --- | --- | --- | --- | --- | --- | --- |
|  | Accuracy | AUC | Accuracy | AUC | Accuracy | AUC | Accuracy | AUC | Accuracy | AUC |
| K = 2 fold  ($\frac{1}{2}$ training $\frac{1}{2}$ testing) | 45.6 % | 0.4618 | 48.5 % | 0.5021 | 53.7 % | 0.5834 | **94.2 %** | **0.9549** | 65.4 % | 0.7724 |
| K = 3 fold  ($\frac{2}{3}$ training $\frac{1}{3}$ testing) | 48.2 % | 0.4722 | 51.5 % | 0.5053 | 56.8 % | 0.6055 | **96.2 %** | **0.9692** | 65.3 % | 0.7752 |
| K = 4 fold  ($\frac{3}{4}$ training $\frac{1}{4}$ testing) | 50.8 % | 0.5119 | 53.4 % | 0.5128 | 57.5 % | 0.6264 | **96.5%** | **0.9712** | 64.5 % | 0.7662 |
| K = 5 fold  ($\frac{4}{5}$ training $\frac{1}{5}$ testing) | 52.7 % | 0.5244 | 55.8 % | 0.5407 | 58.6% | 0.6530 | **95.8%** | **0.9647** | 66.4 % | 0.7754 |
